# Strongly Luminescent Composites Based on Carbon Dots Embedded in a Nanoporous Silicate Glass

**DOI:** 10.3390/nano10061063

**Published:** 2020-05-30

**Authors:** Evgeniia A. Stepanidenko, Pavel D. Khavlyuk, Irina A. Arefina, Sergei A. Cherevkov, Yuan Xiong, Aaron Döring, Georgii V. Varygin, Dmitry A. Kurdyukov, Daniil A. Eurov, Valery G. Golubev, Mikhail A. Masharin, Alexander V. Baranov, Anatoly V. Fedorov, Elena V. Ushakova, Andrey L. Rogach

**Affiliations:** 1 Center of Information Optical Technologies, ITMO University, 49 Kronverkskiy Pr., 197101 St. Petersburg, Russia; stepanidenko.e@mail.ru (E.A.S.); khavlyuk.stepnogorsk@gmail.com (P.D.K.); irina-arefina97@mail.ru (I.A.A.); s.cherevkov@corp.ifmo.ru (S.A.C.); a_v_baranov@yahoo.com (A.V.B.); a_v_fedorov@inbox.ru (A.V.F.); andrey.rogach@cityu.edu.hk (A.L.R.); 2 Department of Materials Science and Engineering, and Centre for Functional Photonics (CFP), City University of Hong Kong, 83 Tat Chee Avenue, Kowloon, Hong Kong, China; yuanxiong3-c@my.cityu.edu.hk (Y.X.); adoering2-c@my.cityu.edu.hk (A.D.); 3 Interdisciplinary Resource Center for Nanotechnology, St. Petersburg State University, 7/9 Universitetskaya nab., 199034 St. Petersburg, Russia; g.varygin@spbu.ru; 4 Laboratory of Amorphous Semiconductors, Ioffe Institute, 26 Politekhnicheskaya Str., 194021 St. Petersburg, Russia; kurd.gvg@mail.ioffe.ru (D.A.K.); edan@mail.ru (D.A.E.); golubev.gvg@mail.ioffe.ru (V.G.G.); 5 Department of Physics and Engineering, ITMO University, 49 Kronverkskiy Pr., 197101 St. Petersburg, Russia; mikhail.masharin@metalab.ifmo.ru

**Keywords:** composite materials, carbon dots, nanoporous silicate glass, photoluminescence

## Abstract

Luminescent composites based on entirely non-toxic, environmentally friendly compounds are in high demand for a variety of applications in photonics and optoelectronics. Carbon dots are a recently developed kind of luminescent nanomaterial that is eco-friendly, biocompatible, easy-to-obtain, and inexpensive, with a stable and widely tunable emission. Herein, we introduce luminescent composites based on carbon dots of different chemical compositions and with different functional groups at the surface which were embedded in a nanoporous silicate glass. The structure and optical properties of these composites were comprehensively examined using electron microscopy, Fourier transform infrared transmission, UV-Vis absorption, and steady-state and time-resolved photoluminescence. It is shown that the silicate matrix efficiently preserved, and even enhanced the emission of different kinds of carbon dots tested. The photoluminescence quantum yield of the fabricated nanocomposite materials reached 35–40%, which is comparable to or even exceeds the values for carbon dots in solution.

## 1. Introduction

Luminescent carbon dots (CDs) are being extensively studied due to their attractive optical properties, simple fabrication methods and perspectives for bio- and photonic applications [1,2]. CDs can be thought of as colloidal nanoparticles with emissive centers incorporated into the carbon-based core, typically with sizes below 10 nm, and with different chemical functional groups at the surface. The nature of emission centers of CDs strongly depends on their chemical composition and, hence, precursors used during their synthesis [3]; they may originate from polycyclic aromatic hydrocarbon (PAH) [4,5], organic dyes [6,7], or heteroatom-containing groups [8,9], which are located within the CD core and/or at their surface [10,11]. By changing the type of precursors employed in the synthesis of CDs and varying the solvents and pH of the reaction medium, the chemical composition of both the core and the surface of the CDs can be adjusted [12,13,14,15]. Thus, for CDs synthesized from one of the most common precursors, citric acid, their surface is rich with C=O, –OH, and –COOH groups. There are several advantages of CDs as compared to organic dyes, namely higher photostability and resistance to photobleaching [16,17,18]. Different from widely studied semiconductor quantum dots such as CdSe and PbSe, which include toxic heavy metals, CDs are non-toxic and thus more suitable for use in biological systems [2,19,20,21,22,23].

For use in photonics and optoelectronics, the availability of solid-state composites based on luminescent CDs is in high demand; such luminescent composite materials have already been implemented for data encryption [24,25,26], in light-emitting diodes (LEDs) [24,27,28,29,30], and in luminescent solar concentrators [31]. However, photoluminescence (PL) properties of CDs may alter significantly on transition from solution to solid-state: both the PL profile and intensity may experience undesirable changes [32], even up to complete quenching [30,33]. Similar to other chromophores, the physical origin behind these changes is determined by self-quenching and/or non-radiative energy transfer within aggregated nanoparticles [34,35]. Therefore, there is an on-going search for suitable matrices which can be conveniently used for the formation of solid composite materials with CDs, and at the same time can preserve or even enhance their PL properties. The host materials include polymers [24,28,36,37], silica [25,30,38,39,40], zeolites [41], potash alum [42], starch [43], etc. For use in photonic and light-converting devices, matrices should be transparent in the visible range, which is achieved for the polymers and glasses. The use of polymer matrices also led to phosphorescent composites with a long lifetime in their excited state [24,36,37]. Ren et al. [44] fabricated highly luminescent CD-based glasses with a high PL quantum yield (QY) of ~40%. The use of polyvinyl butyral as a matrix made it possible to prevent the self-quenching of CDs, which were then used for white LEDs [45]. Sargent’s group [27] fabricated blue LEDs based on CDs incorporated into poly(N-vinyl carbazole) matrix, with a high electroluminescence QY of 4%. Other popular host matrices for CDs are inorganic silicon-based glasses, for which PL QYs as high as 70% were reported [40]. It was shown that phosphorescent composites can be formed via covalent bonding of CDs with silica matrices [25,38].

Despite the intense research in this field, there is still a lack of knowledge about the interactions between silica glasses and different kinds of CDs, and there is still plenty of room in the search for suitable and universal matrices for preserving and even enhancing the optical properties of CDs. In this work, we developed the fabrication method towards luminescent composites based on CDs embedded in a nanoporous silicate glass (NSG), by employing the capillary method. To demonstrate that this matrix is indeed universal and is suitable for infiltration of different types of CDs, we used CDs prepared from various chemical precursors using the solvothermal method with different molecular groups at the surface. A thorough comparison of the optical properties of resulting CD@NSG composites with colloidal dispersions of CDs showed that the use of NSG as a host matrix indeed resulted in the perfect preservation, or even enhancement of the emission of CDs. These findings are of importance for the future design of active materials based on CDs for photonic devices.

## 2. Materials and Methods

### 2.1. Experimental Setup

Absorption and photoluminescence (PL) spectra were collected on a spectrophotometer UV-3600 (Shimadzu, Kyoto, Japan) and on spectrofluorometers FP-8200 (Jasco, Tokyo, Japan) and FLS920P (Edinburgh Instruments, Livingston, UK), respectively. PL QY was measured with an integrating sphere (Labsphere, North Sutton, NH, US), using a 405 nm diode laser as an excitation source. Fourier transform infrared (FTIR) spectra were collected on an infrared spectrophotometer Tenzor II (Bruker, Billerica, MA, US). Scanning electron microscopy (SEM) images were collected on a Merlin instrument (Zeiss, Oberkochen, Germany). Optical imaging has been performed on a confocal microscope LSM-710 (Zeiss, Oberkochen, Germany) equipped with 20× objective (NA  =  0.4) and a 405 nm diode laser as an excitation source. Time-resolved PL measurements were performed on a confocal microscope MicroTime 100 (PicoQuant, Berlin, Germany) equipped with 100× objective (NA  =  0.95) or 3× objective (NA  =  0.1), and a 405 nm pulsed diode laser. PL decay curves were fitted by a biexponential function: $I\left(t\right)=I_{0}+{\;A}_{1}e^{-t/\tau_{1}}+{\;A}_{2}e^{-t/\tau_{2}}$. The average PL lifetime has been calculated as $\langle \tau \rangle =\sum^{\;}A_{i}{\tau_{i}}^{2}/\sum^{\;}A_{i}\tau_{i}$.

### 2.2. CDs Synthesis

Two kinds of CDs with emission in blue and green spectral region, subsequently denoted as CD-1 and CD-2, were chosen for the formation of composite materials. CD-1 and CD-2 were synthesized by the solvothermal method described elsewhere [4,10]. Briefly, for CD-1, 1.05 g of citric acid and 335 μL of ethylenediamine were dissolved in 10 mL of water to form a transparent solution. For CD-2, 1.0 g of citric acid and 2.0 g of urea were dissolved in 10 mL of dimethylformamide (DMF). Each solution was heated up in a Teflon-lined autoclave at 160 °C for 6 h. After cooling down to room temperature, the solutions were transferred into a dialysis bag (molecular weight cut-off: 14000) and dialyzed against deionized water for 1 day. After dialysis, the CD solutions were concentrated to ~25 mg mL^−1^ and stored at ambient conditions. According to literature reports, the size of CD-1 is in the range of 2–5 nm [4,46], and that of CD-2 is in the range of 4–10 nm [47].

Alongside CD-1 and CD-2, another two types of CD samples were synthesized, denoted as CD-3 and CD-4. To produce CD-3, 1 g of phloroglucinol has been used as a carbon source, which was dissolved in a 10 mL mixture of DMF and N-methylformamide (1/1 by volume), followed by heating for 6 h at 160 °C in an autoclave. CD-4 were obtained by dissolving 1 g of phloroglucinol and 2 g of thiourea in a 10 mL mixture of DMF and N-methylformamide (1/1 by volume), followed by heating for 6 h at 190 °C in an autoclave. After cooling down to room temperature, the solutions were purified from unreacted precursors and molecular fluorophores by the same dialysis procedure as for CD-1 and CD-2.

### 2.3. Fabrication of CD@NSG Composites

Porous NSG matrices were fabricated by leaching of sodium-borosilicate glasses as reported in Ref. [48], followed by their annealing at 550 °C in the ambient atmosphere. According to the results of nitrogen porosimetry, the specific surface area and the pore volume of the NSG were 62 m^2^ g^−1^ and 0.15 cm^3^ g^−1^, respectively. An adsorption-structural analysis was made with an ASAP 2020 analyzer (Micromeritics, Communications Drive Norcross, GA, US) at a temperature of 77 K, in which the specific surface area was calculated by the Brunauer–Emmett–Teller method and the pore size distribution was found by the Barrett–Joyner–Halenda method. Average pore size was estimated as 9.3 ± 2.2 nm with the total pore volume of ~15%. The procedure of the impregnation of the NSG matrix with CDs is shown in Scheme S1 (Supplementary Materials). Immediately before impregnation, NSG was annealed in a vacuum oven at 200 °C for 1.5 h, and cooled down to room temperature under ambient conditions. A piece of NSG was completely covered by a droplet of CD solution, which was infiltrated into NSG’s pores by capillary force. Then, the solvent was gently removed by evaporation in an electric stove at 50 °C, and the composites designated hereafter as CD@NSG were obtained. Similarly, NSG samples impregnated with organic molecules instead of CDs were fabricated for reference, and were designated as AA@NSG and C@NSG, where AA stands for 2-aminoacridone and C stands for coronene molecules, respectively.

## 3. Results and Discussion

**Figure 1:**
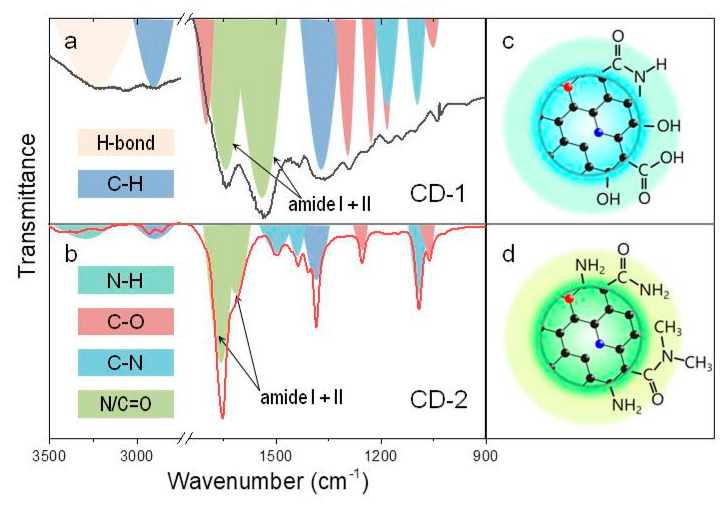
FTIR spectra with respective chemical bond assignments for (**a**) CD-1 and (**b**) CD-2. Schematic presentations of the surface functional groups for CD-1 and CD-2 are provided in (**c**,**d**), respectively. The color used for the CD cores represent their emission; graphitic and pyrrolic nitrogen atoms in the carbon network (shown in black) are given as red and blue circles, respectively.

As already mentioned above, by varying the type of precursors used in the synthesis, different kinds of CDs with variable optical properties and surface chemical composition can be obtained. In Figure 1, Fourier transform infrared (FTIR) spectra of CD-1 and CD-2 are shown. The most intense bands observed in FTIR spectra at 1660–1500 cm^−1^ for both CDs samples are attributed to carboxylic acid derivatives, namely amide bands of R–N/C=O groups, shown by the green area in Figure 1. The higher frequency absorption band (amide I) is attributed to the C=O stretching mode, while the band with lower frequency (amide II) is due to N-H bending. The positions of these two bands suggest that the CD-1 surface contains mostly secondary amides, while the CD-2 surface contains mostly primary amides. This is also supported by comparing the intensity of N-H stretching bands in the 3100–3300 cm^−1^ region, which are more pronounced for the CD-2 sample. The FTIR spectrum of CD-1 has a set of peaks corresponding to C–O stretching: 1700 and 1240 cm^−1^ can be attributed to the carboxyl group, 1290 and 1180 cm^−1^—to C(=O)–O, and 1050 cm^−1^—bending vibration of C–O–C [49]. The band at 1180 cm^−1^ can also belong to the aliphatic C-N stretching. The peak at 1100 cm^−1^ can be associated with C-N-H stretching in the ring. A broad band at 3300 cm^−1^ is typical for H-bonding (polymeric hydrogen-bonded envelope). Similar set of peaks in FTIR spectra was observed for CDs synthesized from citric acid and ethylenediamine [4,6,50], where those bands were attributed to both C–O groups and aromatic structures of molecular fluorophores. X-ray photoelectron spectroscopy (XPS) study revealed the abundance of carboxylic and hydroxyl groups together with nitrogen doping of the CD’s core [4,6,50]. The FTIR spectrum of CD-2 has peaks at 1450, 1405, and 1090 cm^−1^ which can be attributed to the C-N stretching mode. The peaks at 1260 and 1060 cm^−1^ can be associated with C–O bonds [49] in phenol compounds, or =C–O–C groups and bending vibration of C–O–C groups indicating the presence of carbohydrates, respectively. According to XPS measurements reported in Refs. [51,52], which were performed on CDs produced from citric acid and urea, they contain C–C, C=C, C–N, and less C–O groups, with a significant amount of pyrrolic and graphitic nitrogen atoms. Thus, it can be inferred that the surface of CD-1 is rich in C–O and –OH groups, whereas the surface of CD-2 mostly has N-H and C–H groups, as exemplified in Figure 1c,d, respectively.

**Figure 2:**
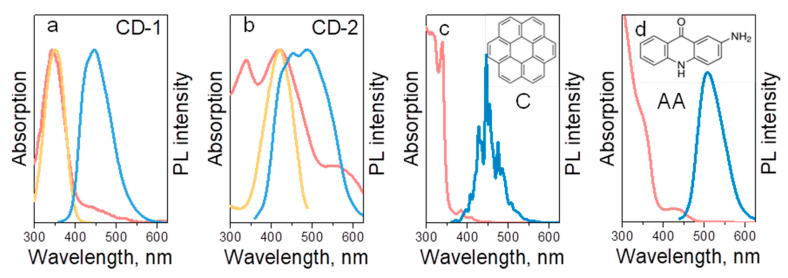
Optical characteristics of (**a**) CD-1 in water, (**b**) CD-2 in methanol, (**c**) coronene (C) in toluene, and (**d**) 2-aminoacridone (AA) in DMF. Absorption spectra are presented in red; PLE spectra (monitored at 450 nm in (**a**), and at 500 nm in (**b**)) are presented in orange; PL spectra excited at 350 nm are provided in blue. Chemical structures of C and AA molecules are provided on the respective frames.

In Figure 2a,b, absorption, PL and PL excitation (PLE) spectra of CD-1 and CD-2 are shown, respectively. PLE spectra were monitored at CD’ PL maxima, namely at 450 nm for CD-1 and at 500 nm for CD-2. CD-1 has one major absorption band at 350 nm, which is perfectly coinciding with the PLE peak and can be attributed to the n-π* optical transition. PL spectrum of CD-1 is centered at 450 nm when excited at 350 nm, with a full width at half maximum (FWHM) of 80 nm and PL QY of 34%. The emission of these CDs is excitation dependent, with PL peak shifting from 440 to 460 and 500 nm when the excitation changes from 350 to 405, and 450 nm, respectively, along with a decrease of PL intensity. In contrast to CD-1, the absorption spectrum of CD-2 contains three peaks at 340, 420, and 570 nm, which can be attributed to the more complex CD structure, including different types of nitrogen doping of CD core [53,54], amino functionalized surface [55], and presence of organic moieties, such as 4-hydroxy-1*H*-pyrrolo [3,4-*c*]pyridine-1,3,6(2*H*,5*H*)-trione, as claimed in Ref. [7]. The PL band excited at 350 nm is centered at 470 nm, with a FWHM of 154 nm and PL QY of 17%. PLE peak position monitored at 500 nm is located at 420 nm and corresponds to the n-π* optical transition as well. The excitation-emission dependence for CD-2 is even more pronounced than for CD-1: the PL peak shifts from 470 to 530 nm when excitation is changed in the range from 350 to 450 nm, and FWHM decreases from 155 to 85 nm with the excitation wavelength. For excitation above 500 nm, the PL intensity of CD-2 drops significantly. Thus, the difference in optical transitions of these two CD samples can be ascribed to the different degree of nitrogen doping of the core and presence of molecular groups at CD’s surface, which according to literature reports results in a redshift of both absorptions and emission transitions [53,54,55].

To understand how interactions between the CD’s and matrix’s surface groups affect the optical properties of composites, organic molecules with different molecular structures and emission were chosen as reference samples. Coronene (abbreviated as C) is an example of a polycyclic aromatic hydrocarbon (PAH), which is a nonpolar, lipophilic molecule emitting in the blue region. According to the literature [4,5,10,46], PAH have been identified as light-emitting units of CDs. In Ref. [4], it was shown that the emission of CDs can be presented as a sum of PL spectra from the PAH molecules of different size. In Ref. [10], it was shown that those PAH molecules and their derivatives are located in the amorphous carbon network forming CD. The second organic molecule (2-aminoacridone, abbreviated as AA) is a green-emitting organic dye containing both primary and secondary amines as well as ketone groups, which can interact with each other and with the matrix. The chemical structures of coronene and AA molecules, and their absorption and PL spectra measured in good solvents toluene and DMF, respectively are provided in Figure 2c,d. The absorption and PL spectra of coronene contain a set of multiple peaks typical for aromatic molecules [56], with the main PL maximum centered at 450 nm, and an envelope FWHM of 65 nm. The absorption spectrum of 2-aminoacridone contains peaks at 350 and 435 nm; its PL spectrum is centered 515 nm with FWHM of 70 nm.

**Figure 3:**
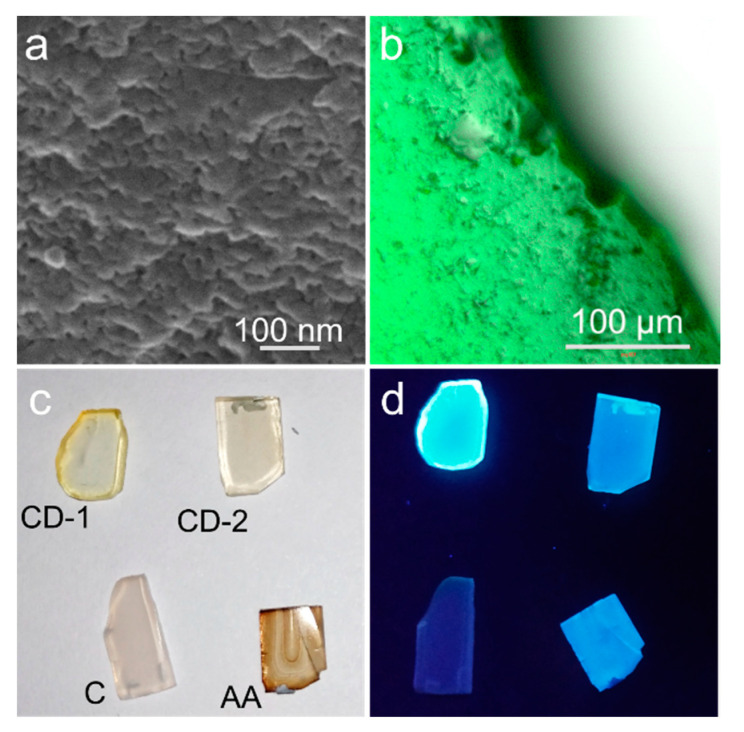
(**a**) SEM image taken from the edge of CD-1@NSG composite. (**b**) Overlay of the microscopic images of the CD-1@NSG composite in transmission and luminescent channels in artificial (green) color. Photographs of the CD-1@NSG, CD-2@NSG, C@NSG and AA@NSG samples under (**c**) visible and (**d**) UV light.

SEM image of the CD-1@NSG composite taken from the edge view demonstrates porous structure of NSG (Figure 3a). An overlay of microscopic images of this composite collected in transmission and luminescent channels (Figure 3b) confirms that the CDs are distributed evenly within the NSG volume. Figure 3c shows that composites have different transparency, with an optical density decreasing in the series of the samples AA@NSG, CD-1@NSG, CD-2@NSG, and C@NSG. This observation suggests the difference of the penetration rate and the resulting concentration of luminophores (CDs and dyes), inside the NSG volume. This can be related to the overall surface charge of the NSG pore surface and different types of surface molecular groups on the CDs or dyes employed. CD@NSGs composites were rather transparent over the visible spectral range from 450 to 800 nm, with an optical density less than 0.1 at the absorption peak for the NSG matrix as thick as 1–1.5 mm.

**Figure 4:**
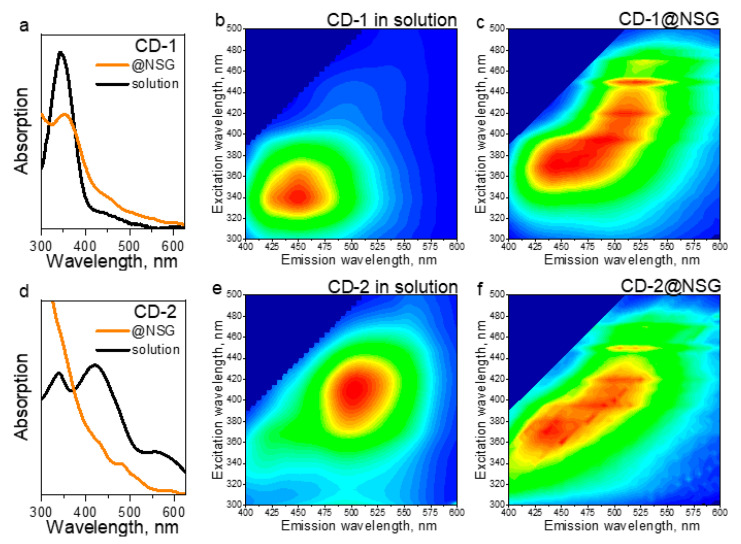
(**a**,**d**) Absorption spectra of CD-1 and CD-2 in solution (black) and embedded in NSG (orange). PLE-PL maps of (**b**,**c**) CD-1 and (**e**,**f**) CD-2 in solution (**b**,**e**) and in NSG (**c**,**f**).

Figure 3d demonstrates that all four CD@NSG and dye@NSG composites possess bright emission, which is strikingly different from the weakly luminescent film samples obtained by a drop-casting of CD-1 solutions onto the glass slides, as shown in Figure S1 in Supplementary Materials. In the latter case, CDs were assembled in dense-packed ensembles during the solvent evaporation, with a formation of characteristic “coffee rings” and strong accompanying PL quenching [57,58,59]. The optical parameters of CD@NSG composites, compared with those of CDs solutions, are summarized in Table 1, which also provides calculated average PL lifetimes. For the CD-1@NSG, the absorption spectrum was very much similar to the one of CD-1 in solution (Figure 4a), with some relative increase of optical density in the 400–550 nm region. At the same time, the radiative transitions underwent noticeable changes: from the PLE-PL maps shown in Figure 4b,c, it can be seen that the contribution of surface states in the 400–500 nm spectral region to the emission increased, which well coincided with the change of the absorption spectra. The PL band of CD-1@NSG shifted from 450 to 510 nm and became broader, showing a stronger excitation dependence compared to the CD-1 solution. This shift of emission from blue to the green spectral region is similar to CDs synthesized from polyacrylic acid and ethylenediamine with –COOH groups on the CD’s surface and then covered by the silica [38]. In the case of CD-2@NSG, the absorption spectrum had two shoulders at 340 and 430 nm, which are related to absorption peaks of the CD-2 solution, and an additional peak at 480 nm (Figure 4d). Comparison of the PLE-PL maps (Figure 4e,f) of the CD-2 solution and CD-2@NSG shows that the PL intensity of composite material increased in the 420–460 nm spectral region, with a more pronounced excitation-dependence of the PL spectrum as was observed for CD-1@NSG. The PLE spectrum of CD-2@NSG showed gradual decrease of the intensity upon increasing wavelength (Figure 4d,f). A subtle shift of 10 nm of the PL peak position with increased FWHM from 90 to 115 nm of CD-2@NSG compared to the CD-2 solution agreed well with the reported composite material based on CDs with amine-rich surface [25], where it was shown that the PL spectrum of composite material in the green spectral region almost coincided with the pristine CDs.

We then analyzed optical spectra of composite samples based on the two dye molecules, AA and C. The optical parameters of AA@NSG drastically changed as compared with solution sample: both absorption and PL bands blue-shifted by 30–40 nm together with a significant decrease in PL intensity (Figure S2, Supplementary Materials). As for the C@NSG composite, the absorption and PL band positions remained the same, but with a decreased PL intensity, suggesting that the coronene molecules did not interact with the charged surface of NSG. The PL QYs decreased to 2% for both AA and C molecules embedded in NSG, which may be due to aggregation of those smaller molecules inside the NSG pores, which is expected would lead to the emission quenching [35,60,61]. This effect was even more pronounced for the AA@NSG composite, where it may additionally be caused by formation of H-aggregates, resulting in the observed blueshift of the emission band (Figure S2c).

We then analyzed and compared PL QYs and the average PL lifetimes of the two CD-based composites and their respective CDs in solutions. The PL lifetime of the CD-1 decreased from 12.3 ns in aqueous solution to 7.5 ns in NSG, while their PL QYs were almost the same, 34–35% (Table 1). PL lifetimes of CD-1 in solution and in NSG had their maximal value observed at positions of the respective PL peaks (Figure S3, Supplementary Materials), which means that the spectral dependence of PL lifetimes almost coincided with their PL spectral profile. For CD-2@NSG, no significant changes in optical parameters were observed (Table 1), while PL QYs of CD-2 has increased almost 2-fold—from 17% in solution to 40% in the NSG. This could be due to interactions of positively charged groups on the CD-2 surface, –NH_2_ (Figure 1d), with the negatively charged pore’s surface of NSG, as was reported in Ref. [62] for a similar NSG, resulting in an increased PL QY. This is also in good agreement with the reported observations from Ref. [36] for the same type of CDs embedded in PVA films: the PL QY increased almost 3 times with respect to the CD aqueous solution, which was explained by the successful passivation of the nonradiative defect states and CDs surface. Since CDs are complicated objects, no obvious relation between their PL lifetime and PLQY was observed. This fact can be explained by taking into account a possible energy transfer both between luminescent centers within one CD and between CDs in the glass pores, and interaction of CD surface groups with the matrix. It has previously been shown that silica matrix can protect CDs from self-quenching and external quenching [34,63]. At the same time, in Ref. [38] it was shown that the increase of PLQY was accompanied with a slight decrease of PL lifetime, which was ascribed to the formation of C–O–Si bonds in a silica matrix, which passivated the CD surface. This possible scenario is in agreement with the findings of our FTIR analysis (Figure 1), which shows that CD with different surface groups, namely carboxyl and amino groups, interact with silica matrix differently, which affects the optical properties of their composites.

To further demonstrate that the fabrication procedure of luminescent composite materials is indeed universal for different types of CDs and can be conducted from different solvents, we synthesized two other kinds of CD samples. CD-3 was synthesized from phloroglucinol in a solvent mixture of DMF and methylformamide, while CD-4 was produced from a mixture of phloroglucinol and thiourea in the same solvent mixture. The optical spectra of these CDs in their respective solutions are given in Figure S4 in the Supplementary Materials. From the analysis of their FTIR spectra (Figure S5, Supplementary Materials), the synthesized CDs possessed different molecular groups on their surface: the CD-3 surface was rich with –CH_3_, –OH, and amide groups, while the CD-4 surface also contained –NH, C=O, and C=S groups. Both samples also showed a broad band at 3100 cm^−1^, which is attributed to H-bonding. Optical properties of CD embedded into the NSG, such as the absorption and PL peak positions, were well preserved (Figure S6, Supplementary Materials). Importantly, PL signal of all the 4 studied CD@NSG composites remained stable for several months, with only a slight decrease in PL intensity.

## 4. Conclusions

In conclusion, we have developed a fabrication procedure for luminescent composites based on different types of CDs embedded into nanoporous silicate glass. In a stark contrast to the quenched emission of CD films drop-casted directly onto glass slides, the photoluminescence properties of CDs were preserved while embedding them into the glass matrix. The PL QY of the fabricated nanocomposite materials reached 35–40%, which is comparable to or even exceeds the PL QY of CDs in solution. We showed that the developed protocol is suitable for the CDs synthesized from different precursors and, hence, having different functional groups at the surface. We also revealed that the incorporation of organic dyes into the matrix, in contrast to CDs, led to significant quenching of PL, presumably due to the aggregation of smaller dye molecules inside NSG pores. It is worth noting that CD-based composite samples possessed a broad emission spectrum with FWHM of up to 150 nm, which can be advantageous for their implementation as an active medium in white LEDs. These findings are of great importance for the further development of photonic and optoelectronic devices based on CDs.

## Figures and Tables

**Table 1:** Optical parameters of CD-1 and CD-2 samples in solution and in NSG.

Sample	Abs Peak Position, nm		PL Peak Position, nm		PL Lifetime, ns		PL QY, %	
	solution	@NSG	solution	@NSG	solution	@NSG	Solution ex. at 350 nm	@NSG ex. at 405 nm
CD-1	350 ± 2	360 ± 5	450 ± 2	510 ± 5	12.3 ± 0.5	7.5 ± 0.5	34 ± 1	35 ± 5
CD-2	430 ± 2	430 ± 5	500 ± 2	510 ± 5	8.4 ± 0.5	8.7 ± 0.5	17 ± 1	40 ± 5

## Supplementary Materials

The following are available online at https://www.mdpi.com/2079-4991/10/6/1063/s1. Materials; Scheme S1: Fabrication of composites based on CDs infiltrated into the NSG matrices; Figure S1: Photographs of CD-1 drop-casted on a glass slide; Figure S2: Absorption and PL spectra of 2-aminoacridone and coronene measured in solution and in NSG; Figure S3: PL spectra and average PL lifetimes of CD-1 in solution and embedded into NSG; Figure S4: Optical characteristics of CD-3 and CD-4 in methanol; Figure S5: FTIR spectra of CD-3 and CD-4; Figure S6: Absorption and PL spectra of CD-3 and CD-4 in solution and embedded into NSG.
