# Supplementary material for: Strongly Luminescent Composites Based on Carbon Dots Embedded in a Nanoporous Silicate Glass
Source: Nanomaterials (Basel). 2020 May 30;10(6):1063. doi: 10.3390/nano10061063 (PMC7352239; doi:10.3390/nano10061063)
Supplement: Supplementary file 1 [file nanomaterials-10-01063-s001.pdf]

nanomaterials

Article

Strongly Luminescent Composites Based on Carbon Dots Embedded in a Nanoporous Silicate Glass

Evgeniia A. Stepanidenko [1], Pavel D. Khavlyuk [1], Irina A. Arefina [1], Sergei A. Cherevkov [1], Yuan Xiong [2], Aaron Döring [2], Georgii V. Varygin [3], Dmitry A. Kurdyukov [4], Daniil A. Eurov [4], Valery G. Golubev [4], Mikhail A. Masharin [5], Alexander V. Baranov [1], Anatoly V. Fedorov [1], Elena V. Ushakova [1,2,*] and Andrey L. Rogach [1,2]

[1] Center of Information Optical Technologies, ITMO University, 49 Kronverkskiy Pr., 197101 St. Petersburg, Russia; stepanidenko.e@mail.ru (E.A.S.); khavlyuk.stepnogorsk@gmail.com (P.D.K.); irina-arefina97@mail.ru (I.A.A.); s.cherevkov@corp.ifmo.ru (S.A.C.); mikhail.masharin@metalab.ifmo.ru (M.A.M.); a_v_baranov@yahoo.com (A.V.B.); a_v_fedorov@inbox.ru (A.V.F.); andrey.rogach@cityu.edu.hk (A.L.R.)

[2] Department of Materials Science and Engineering, and Centre for Functional Photonics (CFP), City University of Hong Kong, 83 Tat Chee Avenue, Kowloon, Hong Kong, China; yuanxiong3-c@my.cityu.edu.hk (Y.X.); adoering2-c@my.cityu.edu.hk (A.D.)

[3] Interdisciplinary Resource Center for Nanotechnology, St. Petersburg State University, 7/9 Universitetskaya nab., 199034 St. Petersburg, Russia; g.varygin@spbu.ru

[4] Laboratory of Amorphous Semiconductors, Ioffe Institute, 26 Politekhnicheskaya Str., 194021 St. Petersburg, Russia; kurd.gvg@mail.ioffe.ru (D.A.K.); edan@mail.ru (D.A.E.); golubev.gvg@mail.ioffe.ru (V.G.G.)

[5] Department of Physics and Engineering, ITMO University, 49 Kronverkskiy Pr., 197101 St. Petersburg, Russia; mikhail.masharin@metalab.ifmo.ru

* Correspondence: elena.ushakova@itmo.ru

Supporting Information

Materials

Materials. Citric acid (≥99,5 %), ethylenediamine (≥99,5 %), N, N'-dimethylformamide (99,8 %), urea (BioReagent), thiourea (99 %), pholoroglucinol (99 %), 2-aminoacridone (≥98 %), coronene (99%) were purchased from Sigma-Aldrich, Inc. N-methylformamide (99,5 %) was purchased from “Chemical Line”, Russia; toluene (99,5%) was purchased from “Vecton”, Russia. All chemical reagents were used as received. Ultrapure water (Milli-Q water) was used throughout the experiments.

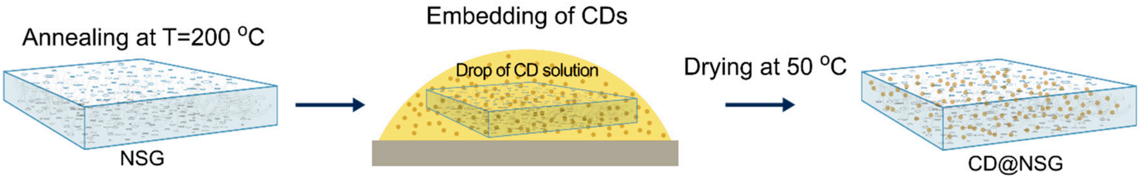

Scheme S1. Fabrication of composites based on CDs infiltrated into the NSG matrices (CD@NSG).

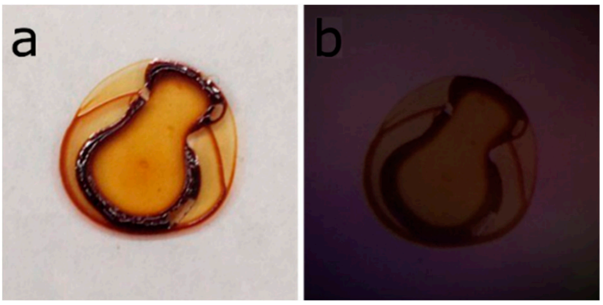

Figure S1. Photographs of CD-1 drop-casted on a glass slide, taken under (a) visible and (b) UV light.

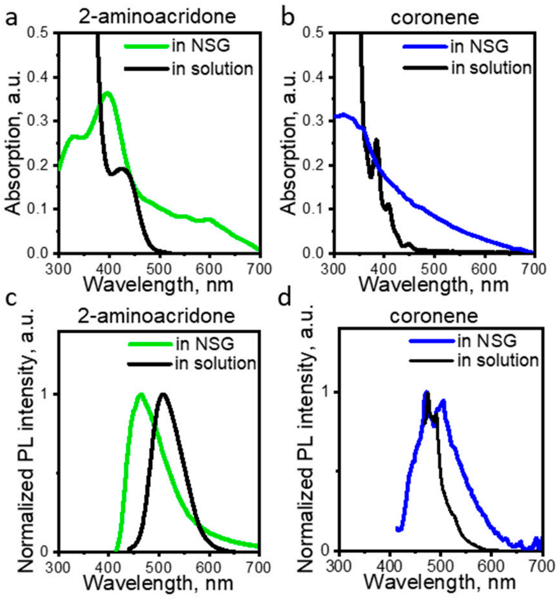

Figure S2. (a,b) Absorption and (c, d) PL spectra of (a, c) 2-aminoacridone and (b, d) coronene measured in solution and in NSG. The excitation wavelength was 405 nm for all PL spectra.

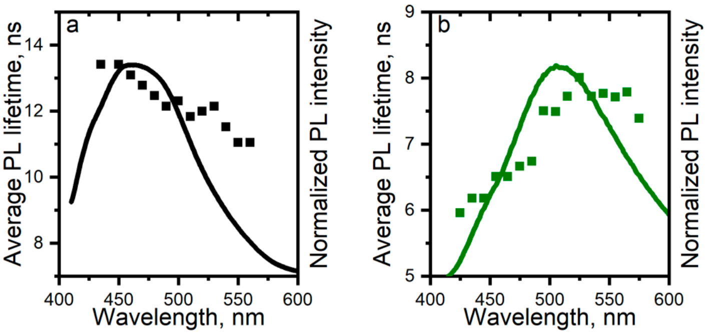

Figure S3. PL spectra (solid lines) and average PL lifetimes (squares) of the CD-1 in solution (a) and embedded into NSG (b).

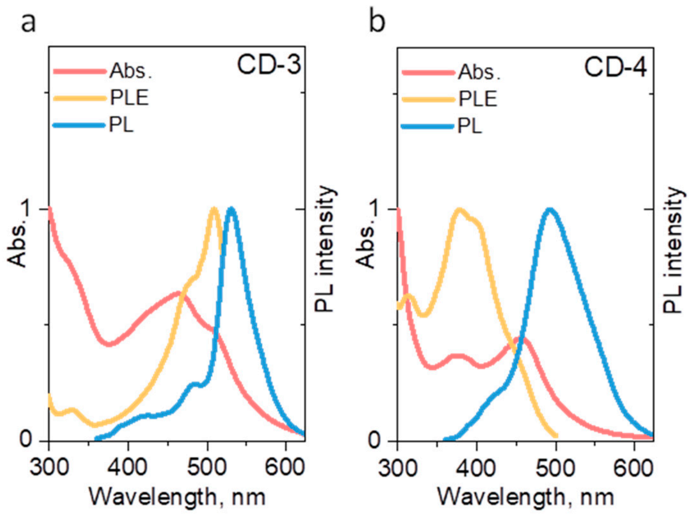

Figure S4. Optical characteristics of (a) CD-3 and (b) CD-4 in methanol. Absorption spectra are presented in red; PLE spectra (monitored at 530 nm in a, and at 500 nm in b) are presented in orange; PL spectra excited at 350 nm are provided in blue.

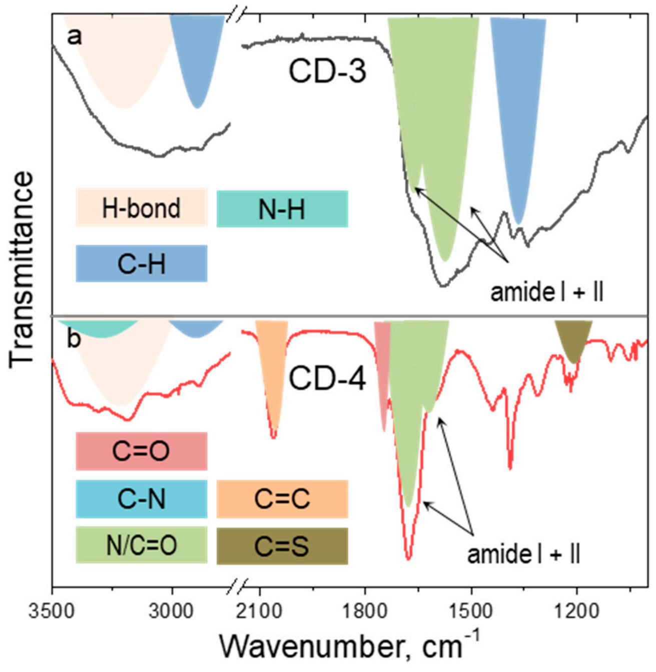

Figure S5. FTIR spectra of (a) CD-3 and (b) CD-4.

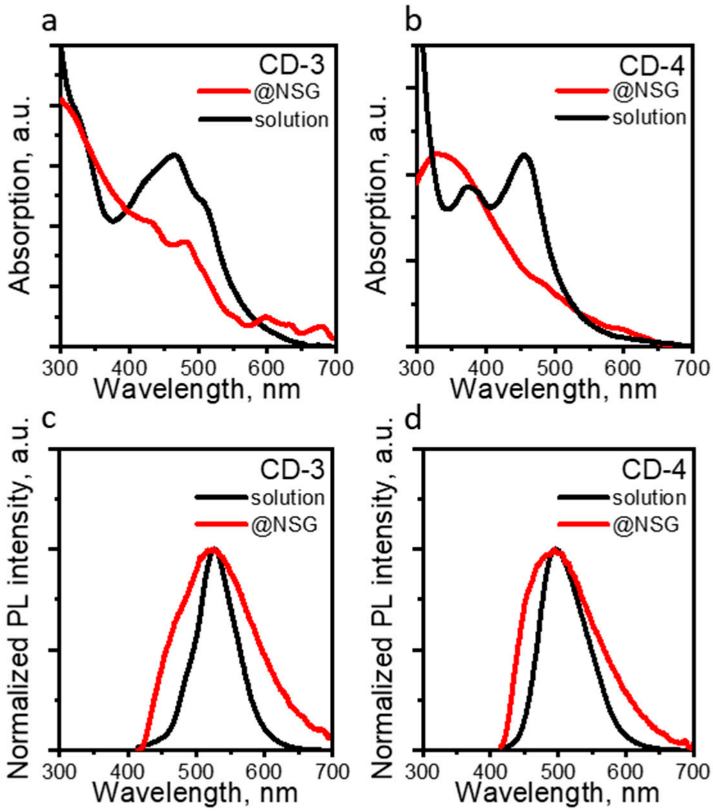

Figure S6. (a, b) Absorption and (c, d) PL spectra of (a, c) CD-3 and (b, d) CD-4 in solution (black lines) and embedded into NSG (red lines). Excitation wavelength was 405 nm for all PL spectra.

© 2020 by the authors. Submitted for possible open access publication under the terms and conditions of the Creative Commons Attribution (CC BY) license (http://creativecommons.org/licenses/by/4.0/).
